# Total knee arthroplasty for Ochronosis induced knee arthropathy. Case report

**DOI:** 10.1016/j.ijscr.2020.06.005

**Published:** 2020-06-11

**Authors:** Mohamed Al Ateeq Al Dosari, Aissam Elmhiregh, Safa Abulhail, Elhadi Babikir, Shamsi Abdul Hameed

**Affiliations:** Hamad Medical Corporation, Doha, Qatar

**Keywords:** TKA, total knee arthroplasty, OA, osteoarthritis, Total knee arthroplasty, Ochronosis, Alkaptunurea, Black bone disease, Homogentisic acid

## Abstract

**Introduction:**

Alkaptunurea is a rare metabolic disorder with autosomal recessive genetic pattern in transmission, it is characterized by accumulation of hemogenistic acid in the tissues due to deficiency of homogentisate 1,2 dioxygenase activity. Characteristically, affected patient will have dark urine and blackish discoloration of connective tissue, especially cartilage and bone and hence it is known as black bone disease.

**Presentation of the case:**

The reported case is for 49 years old gentleman, known to have hypertension, hypothyroidism and Alkaptunurea. He presented to our facility with long standing bilateral knee pain (more in the left) with difficulty in doing daily activities. He presented to our tertiary facility after failure of previous treatment measures.

The patient underwent uncomplicated Robotic assisted total knee arthroplasty that resulted in significant improvement of his pain and function.

**Conclusion:**

Knee arthroplasty is a reliable and reproducible modality in treating Ochronotic knee arthropathy and it would result in pain reduction and better function of patients with such condition.

## Highlights

• Ochronosis induced osteoarthritis of the knee.
• Clinical features and intraoperative evaluation of Ochronosis induced osteoarthritis.
• Indication for arthroplasty in Ochronosis induced osteoarthritis.
• Total knee arthroplasty 1 year results in Ochronosis induced osteoarthritis.
• Functional results, pain score and satisfaction at 1 year after Arthroplasty for Ochronosis induced knee osteoarthritis.

## 1 Introduction

Alkaptunurea is a rare metabolic disorder with autosomal recessive genetic pattern in transmission, it is characterized by accumulation of homogentisic acid in the tissues due to deficiency of homogentisate 1,2 dioxygenase activity. Characteristically, affected patients will have dark urine and blackish discoloration of connective tissue, especially cartilage and bone and hence it is known as black bone disease [1,2].

Ochronosis is defined as blackish discoloration of connective tissue like skin over cartilage or sclera. HGD gene mutation is causing factor which leads to lack of the functional level of the enzyme responsible for breakdown of homogentisic acid. During infancy, Alkaptunurea is usually asymptomatic and can be noticed by blackish discoloration of urine in diapers after long exposure to air [3,5].

Ochronosis symptoms appears during adulthood after prolonged accumulation of homogentisic acid in the tissues like joints, tendons, ligaments and bone. Inflammatory arthritis and joints stiffness would affect large joints like knees and hips, spinal ankylosis and kyphosis could occur due to disc calcification [2].

## 2 Presentation of the case

The reported case is for a 49 years old Indian gentleman, known to have hypertension, hypothyroidism and Alkaptunurea. He presented first to our tertiary center outpatient clinic in May 2018 complaining of long-standing bilateral knee pain which was more in the left side and chronic low back pain. He has been followed by a senior spine surgeon and the patient was managed well conservatively, regarding his knees pain, different treatment modalities were tried and they never relieved the patient’s symptoms. Long term physiotherapy, life style modifications were all tried with minimal effect. In 2013 and 2016, the patient underwent arthroscopic debridement for right and left knees respectively in other facility and temporary relief was achieved. The patient is married with negative past surgical history apart from what has been mentioned, not doing sport, irrelevant drug history and not smoker. He also had no family history of similar condition.

**Fig. 1:**
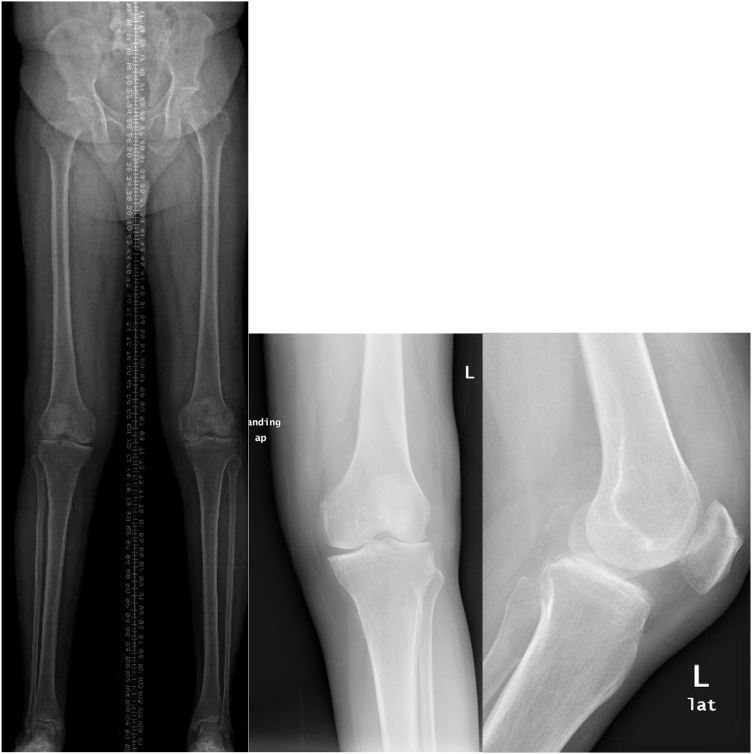
Anteroposterior and lateral views of both knees and long film view.

Upon presentation, the patient had difficulty in ambulating for a distance due to his knees pain primarily, while his knee examination showed severe joint line tenderness and limited range of motion from 10 to 120°, his standing knees X-rays were significant for severe tricompartmental osteoarthritis and correctable valgus deformity of 5°. The knee was stable in anteroposterior and varus-vulgus translation (Figure 1).

**Fig. 2:**
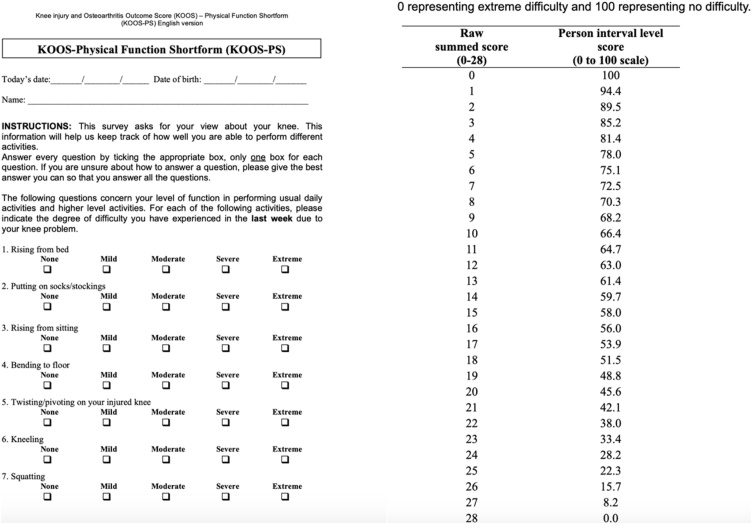
KOOS-PS score and interpretation.

The patient scaled his knee pain as 9/10 on pain visual analogue scale and his function scored very low (28.2) on the Knee Injury and Osteoarthritis Outcome Score – physical function short form (KOOS-PS) [4] (Figure 2).

The patient was counselled for primary total knee arthroplasty as a solution for his knee disability and on July 2018, he underwent uneventful left knee arthroplasty by a senior orthopedic arthroplasty consultant. The surgery was day care and robotic assisted. Intraoperatively, there were significant blackish discoloration of the cartilage and surrounding tissue. However, there were no features of tissue necrosis or bleeding tendency and apart from the discolored tissue, the surgery was straightforward.

**Fig. 3:**
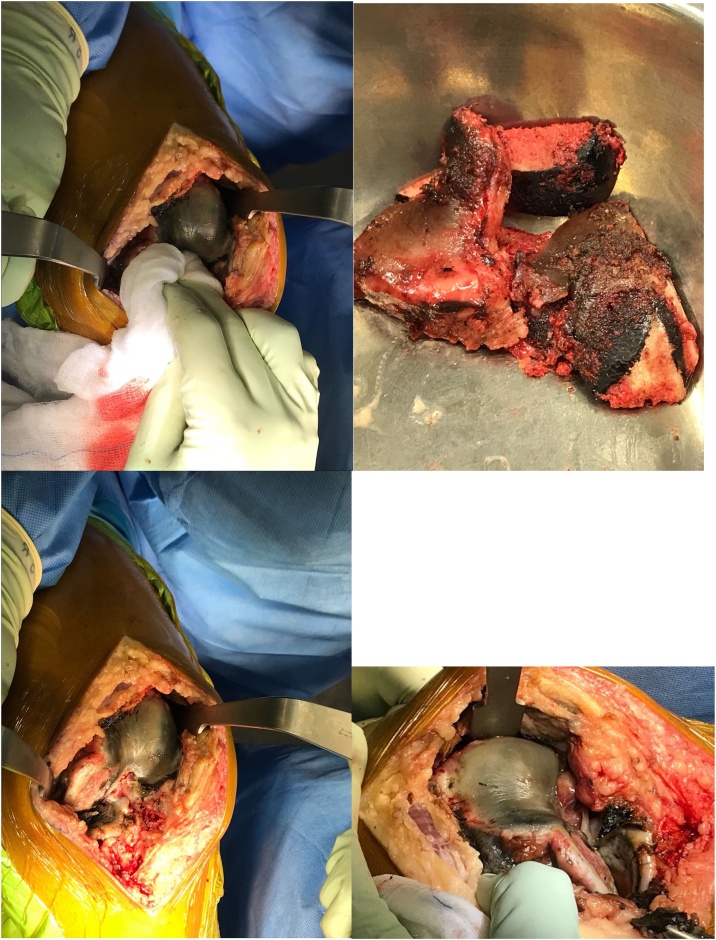
Intraoperative clinical photos of the left knee.

**Fig. 4:**
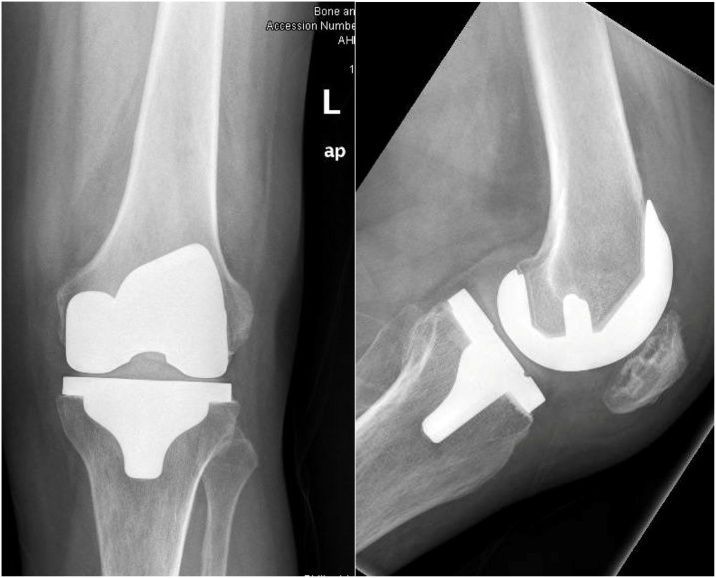
Postoperative X-ray showing cruciate retaining total knee arthroplasty.

Intraoperatively, the knee was stable and both cruciate ligaments were intact and hence a cruciate retaining total knee cemented prosthesis was implanted successfully with patellar resurfacing via medial parapatellar approach. Gentle soft tissue handling and closure with bioabsorbable multifilament suture was done and skin closed with stables and no drain was used (Fig. 3, Fig. 4).

The patient was ambulated immediately postoperatively; however, it was noticed that he had lower pain threshold and consumed maximal postoperative narcotics. the patient was discharged home safely by the end of day. At two weeks follow up, the surgical wound has healed completely with no complications.

Postoperatively, the patient started immediate outpatient accelerated physiotherapy program and at 3 months, he got improved significantly in terms of pain and functional scores. He scored 4/10 on visual analogue scale and 70.3 on KOOS-PS score at 1 year. The patient was satisfied with the procedure and had a silent and painless left knee with almost no symptoms and planning to do the right side soon.

## 3 Discussion

This case report was written in line with SCARE guidelines 2018 [9] and it aimed to add to our understanding of this disease and possible treatments. As most of current evidence on Ochronosis induced knee arthritis are case reports, this case report was supported with functional outcome showing significant improvement after total knee arthroplasty.

Alkaptonuria is a rare systemic metabolic disease that is attributed to the accumulation of homogentisic acid in the tissue. Skeletal system is cardinally affected in terms of articular cartilage degeneration. Hips and knees and the mostly reported joints to be destructed in Ochronosis. Moslavac and his colleagues [7] reported multiple joints involvements in a 70 years old patient that was treated with bilateral total knee arthroplasties and unilateral total hip arthroplasty with acceptable results.

Although the connective tissue in Alkaptunurea patients is burdened with homogentisic acid, arthroplasty seemed to be quite effective and successful treatment as the abnormal tissue appeared to cause no interaction with the prosthesis or the cement. Ulucay et al. [6] reported normal bone healing following initial femur fracture fixation when their reported case underwent total hip arthroplasty for Ochronotic arthropathy. Cement fixation seemed to be as effective for prosthesis fixation as in normal bone [6].

Higher blood loss was reported in arthroplasty surgeries of patients with Ochronosis and that was attributed to the surgeon’s extensive synovectomy [8] in those cases. In our reported case, bleeding was average and no blood product transfusion needed after the surgery. The controversy in our case scenario was whether to sacrifice the cruciate ligament or to use cruciate retaining prosthesis. Tendons and ligaments appeared intact and healthy and we were able to balance the knee without sacrificing the posterior cruciate ligament and hence we used cruciate retaining total knee prosthesis.

## 4 Conclusion

Ochronotic arthropathies are rare conditions that are associated with articular cartilage damage and they can be treated safely and effectively with joint replacement surgery with good functional results, pain improvement and patient satisfaction at 1 year.

## Declaration of Competing Interest

All Authors declare no conflict of interest neither employment, consultancies, stock ownership, honoraria, paid expert testimony, patent applications/registrations, nor grants or other funding.

## Funding

This paper is self-funded by the authors with no sponsorship.

## Ethical approval

This study has been approved by the medical research center in the state of Qatar and the patient of the reported finding has been consented for publication.

## Consent

The patient with reported finding has been consented before writing the case report and publication.

## Author contribution

Dr. Mohamed Al Dosari … study concept, data analysis or interpretation.

Dr. Aissam Elmhiregh … data collection, data analysis or interpretation, writing the paper.

Dr. Safa Abulhail … writing the paper.

Dr. Elhadi Babikir … data analysis or interpretation.

Dr. Shamsi Hameed … data analysis or interpretation.

## Registration of research studies

researchregistry5480.

## Guarantor

Dr. Aissam Elmhiregh.

## Provenance and peer review

Not commissioned, externally peer-reviewed.
